# Outcomes and interventions in the elderly with and without cognitive impairment: a longitudinal study

**DOI:** 10.1590/1980-57642020dn14-040010

**Published:** 2020-12

**Authors:** Anne Caroline Soares da Silva, Juliana Hotta Ansai, Natália Oiring de Castro Cezar, Francisco Assis Carvalho Vale, Julimara Gomes dos Santos, Larissa Pires de Andrade

**Affiliations:** 1 Physiotherapy Department, Universidade Federal de São Carlos – São Carlos, SP, Brazil.; 2 Gerontology Department, Universidade Federal de São Carlos – São Carlos, SP, Brazil.; 3 Medicine Department, Universidade Federal de São Carlos – São Carlos, SP, Brazil.

**Keywords:** continuity of patient care, cognitive dysfunction, Alzheimer disease, aged, continuidade da assistência ao paciente, disfunção cognitiva, doença de Alzheimer, idoso

## Abstract

**Background:**

Clinical follow-up studies are necessary for a better understanding of the evolution of cognitive impairment as well as the development of better assessment and intervention tools.

**Objective:**

To investigate whether older people with preserved cognition (PC), mild cognitive impairment (MCI) and mild Alzheimer's disease (AD) show differences in clinical outcomes and interventions after a 32-month period.

**Methods:**

One hundred twenty-four community-dwelling older people were included and classified in one of three groups (PC, MCI and mild AD). Information on clinical outcomes (deaths, new diagnoses, falls, need for assistance or changes in routine and hospitalizations) and interventions (increased use of medication, physiotherapeutic intervention, practice of physical exercise, etc.) in the 32-month period were collected by telephone or during a home visit on a single day.

**Results:**

Ninety-five participants (35 with PC, 33 with MCI and 27 with AD) were reevaluated after 32 months. The need for assistance/changes in routine was significantly higher in the AD group, especially with regard to basic activities of daily living. Unlike the other groups, the PC group did not show “other diagnoses” (urinary incontinence, prolapse, change in vision or autoimmune disease). No significant differences were found regarding other variables.

**Conclusions:**

Older people with and without cognitive impairment exhibited differences in some clinical outcomes after 32 months, such as need for assistance or changes in their routine and new diagnoses of specific diseases. Therefore, the multidimensionality of geriatric patients should be considered when planning assessments and interventions.

## RESUMO.

**Introdução:**

Estudos com acompanhamento clínico são necessários para um melhor entendimento da evolução do quadro cognitivo e desenvolvimento de melhores estratégias de avaliação e intervenção.

**Objetivo:**

Investigar se idosos com cognição preservada (CP), comprometimento cognitivo leve (CCL) e doença de Alzheimer (DA) leve apresentam diferenças nos desfechos clínicos e intervenções realizadas ao longo de 32 meses.

**Métodos:**

A amostra foi constituída de 124 idosos residentes na comunidade foram incluídos e classificados em um dos três grupos (CP, CCL, DA leve). Informações sobre desfechos clínicos (óbitos, novos diagnósticos, quedas, necessidade de assistência ou mudanças de rotina e hospitalizações) e intervenções realizadas (aumento do uso de medicamentos, intervenção fisioterapêutica, prática de exercícios físicos e outras) foram coletadas, no período de 32 meses, por telefone ou durante visita domiciliar em um único dia.

**Resultados:**

Após 32 meses, 95 idosos (35 CP, 33 CCL e 27 DA) foram reavaliados. A necessidade de assistência/mudanças de rotina foi significativamente maior no Grupo DA, principalmente para as atividades básicas de vida diária. Diferentemente dos outros grupos, o Grupo CP não apresentou “outros diagnósticos” (incontinência urinária, prolapso, alterações visuais e doença autoimune). Nenhuma outra diferença significativa foi encontrada quanto às outras variáveis.

**Conclusão:**

Os idosos com e sem comprometimento cognitivo apresentaram diferenças em alguns desfechos clínicos após 32 meses, como necessidade de assistência/mudanças de rotina e novos diagnósticos de doenças específicas. Assim, a multidimensionalidade do idoso deve ser considerada ao planejar avaliações e intervenções.

## INTRODUCTION

Aging is accompanied by an increase in the occurrence of chronic and degenerative diseases, such as dementia.^1^ The most common type of dementia is Alzheimer's disease (AD),^2^ which is a progressive clinical syndrome, the course of which varies from patient to patient but generally starts with difficulty remembering recent information, apathy and depression.^2^ In advanced stages, major losses occur in motor functions, such as walking, speaking and swallowing.^2^ In contrast, mild cognitive impairment (MCI) is a continuous process between the expected decline resulting from aging and early dementia.^3^ MCI is perceived by the individual, relatives and friends, but does not significantly impair activities of daily living (ADLs).^2^ ^,^ ^4^

Considering the high prevalence of AD and its impact on older people, caregivers, family and society,^2^ longitudinal studies on cognitive aging and its outcomes are needed. The onset of new health issues in older people favors multimorbidity, which is associated with a greater risk of disability, poorer quality of life, hospitalizations and an increased use of health services and medications.^5^ Cognitive impairment contributes to a greater incidence of falls, which can lead to multimorbidity and worse negative outcomes, such as hospitalizations.^6^ Moreover, new clinical outcomes can contribute to increased dependence among older people with AD as well as the progression of the disease, a greater need for care and higher health costs.^7^

Some interventions can influence the evolution of clinical conditions in older people with and without cognitive impairment. Participation in social activities may be a voluntary, selective, compensatory and adaptive change to ensure emotional wellbeing.^8^ Moreover, despite the benefits of pharmacological treatment, the use of medications can lead to greater health costs and adverse events.^9^ It has little impact on cognitive functions in older people with AD and does not benefit those with MCI.^9^ On the other hand, physical exercise can favor cognition in individuals with AD and MCI; it has few adverse effects and there is greater adherence to treatment.^10^ Physical exercise also prevents inactivity, improves neuropsychiatric symptoms and delays the need for assistance regarding ADLs.^11^

It is necessary to monitor the different kinds of interventions. Studies have been conducted to identify the evolution of neuropsychiatric symptoms in individuals with MCI^12^ and AD.^13^ However, few studies have addressed non-psychiatric clinical outcomes, which is essential to the understanding of aging and its association with cognitive impairment.

Thus, the aim of the present study was to investigate whether older people with PC, MCI and mild AD show differences in clinical outcomes (deaths, new diagnoses, falls and consequences, the need for assistance or changes in routine and hospitalizations) and interventions (increased use of medication, participation in social activities, physiotherapeutic intervention, the practice of physical exercise, etc.) after a 32-month period. The hypothesis was that older people with MCI and mild AD would exhibit more negative clinical outcomes and undergo more pharmacological and specific non-pharmacological interventions over time compared to older people with PC.

## METHODS

### Study design and participants

A longitudinal study was conducted involving older people with PC, MCI and mild AD, who were followed up after 32 months (baseline: January to September 2015; follow-up: September 2017 to May 2018). This study was part of the “Brazilian longitudinal study about motor alterations in older people with cognitive disorders (BLSMotorCD)” and received approval from the local human research ethics committee (certificate number: 819.668/2014). All participants or caregivers signed an informed consent form at baseline.

Community-dwelling individuals 65 years of age or older were recruited through pamphlets and folders placed at primary care units as well as through announcements on local radio and TV stations. The inclusion criteria were the ability to walk at least 10 meters without a gait-assistance device and classification in one of the three groups based on cognitive performance. The exclusion criteria were motor sequelae resulting from a stroke, neurological disorders that compromised cognition or mobility, severe audiovisual impairment that would interfere with communication during the evaluations, moderate to advanced AD and the presence of depressive symptoms [assessed using the Geriatric Depression Scale (GDS)].^14^ Individuals who withdrew from the study and those without the possibility of contact during follow-up were also excluded.

The PC group was composed of individuals with a normal score on the Mini-Mental State Examination (MMSE) adjusted for schooling^15^ and without MCI or any criteria for dementia. The diagnostic criteria for MCI were cognitive complaint reported by the subject or an informant (person in contact with the subject at least half of the day four days per week), objective cognitive impairment [score of 0.5 on the Clinical Dementia Rating (CDR)],^16^ normal global cognitive function adjusted for schooling^15^ and preserved function (assessed using Pfeffer's Functional Activities Questionnaire).^17^ The confirmation of AD and other diagnoses was performed by a neurologist and based on the DSM-IV.^18^ Only individuals with a CDR score of 1.0 were included in the AD group.^16^ All scales used to classify the groups were administered at baseline in a closed room by trained assessors (specialists in the field of gerontology).

### Measures

At baseline, the participants underwent an assessment in person by trained assessors with the assistance of an informant in the MCI and AD groups to collect data on socio-demographic characteristics and health information, such as age, sex, schooling, use of medications and presence of diseases. The GDS^14^ and Minnesota Physical Activity and Leisure Questionnaire^19^ were also administered to assess symptoms of depression and determine weekly calorie expenditure, respectively. This information was collected at both baseline and follow-up.

After 32 months, a semi-structured questionnaire was administered either by telephone or during a home visit on a single day. Priority was given to contact via telephone. When necessary, the attempt via telephone was repeated on different days and at different times. However, when this contact was not possible, home visits were made in an attempt to obtain the desired information from the participants. Clinical outcomes (deaths, new diagnoses, falls,^20^ the need for assistance or changes in routine and hospitalizations) and interventions (increased use of medication, participation in social activities, physiotherapeutic intervention, practice of physical exercise, etc.) were collected. Open-ended questions were also posed to identify clinical outcomes or interventions other than those mentioned. All information was collected retrospectively.

### Statistical analysis

Statistical analysis was performed with the aid of *Statistical Package for the Social Sciences* (SPSS), version 22.0, with significance level set at 5% (α=0.05). A descriptive analysis of the data was performed at baseline. The Kolmogorov-Smirnov normality test was applied to all continuous variables to determine the distribution of the data. As most data exhibited non-normal distribution, the Kruskal-Wallis test and post-hoc test (Mann-Whitney test for the comparison of two groups) were used for the quantitative variables and the chi-square association test was used for the categorical variables to evaluate differences between groups.

## RESULTS

**Figure 1:**
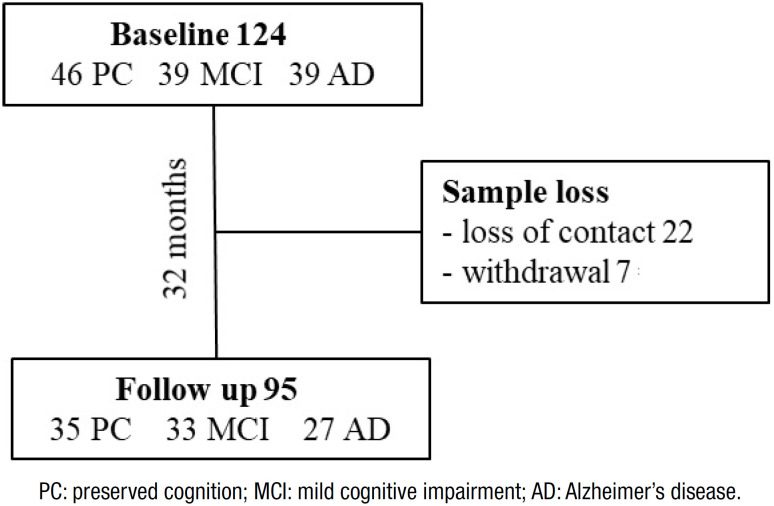
Study flowchart and number of patients.

The baseline sample comprised 46 older people with PC, 39 with MCI and 39 with AD. After 32 months, the final sample consisted of 35 people with PC, 33 with MCI and 27 with AD (Figure 1). In the 32-month period, two (6%) individuals in the MCI group and four (14%) in the AD group died. A total of 29 participants were lost to follow-up.

Regarding characteristics at baseline among the individuals who survived, the AD group was older than the PC and MCI groups. The MCI group included more women than the other groups. The AD and MCI groups used more medications and had more diseases compared to the PC group. Moreover, the AD group had lower calorie expenditure (assessed by the Minnesota Physical Activity and Leisure Questionnaire) than the PC group. The groups were similar regarding the use of psychotropic drugs, schooling and the risk of depression at baseline (Table 1).

**Table 1:** Characteristics of sample at baseline.

	PC group(n = 35)	MCI group(n = 31)	AD group(n = 23)	p-value
Age (years), Md (IQR)	72 (70-78)	75 (70–83)	80 (74–88)*,†	0.003
Female sex, n (%)	20 (57.1)	29 (87.9)	14 (51.9)	0.005
Years of schooling, Md (IQR)	4 (4–8)	4 (3–4)	4.0 (3–11)	0.826
Medication (total number), Md (IQR)	2 (1–5)	5 (3–7.5)*	5.0 (4–6)*	0.0001
Use of psychotropic drugs, n (%)^a^	13 (37.1)	8 (25.8)	13 (56.5)	0.071
Diseases (total number), Md (IQR)	2 (1–3)	3 (2–4)*	3 (2–4)*	0.002
GDS (points) (0–15), Md (IQR)	2 (1–5)	4 (2–5)	3 (2–5)	0.068
Minnesota (points), Md (IQR)	1015.1 (493.5–1999.2)	786.3 (177.2–18885.9)	289.8 (0–1161.1)*	0.033

Md (IQR): median (25–75% interquartile range); n (%): number of individuals (percentage); PC: preserved cognition; MCI: mild cognitive impairment; AD: Alzheimer's disease; GDS: Geriatric Depression Scale; Minnesota: Minnesota Physical Activity and Leisure Questionnaire;

* p<0.05 compared to PC group;

† p<0.05 compared to MCI group;

a percentage of individuals who took medications.

**Figure 2:**
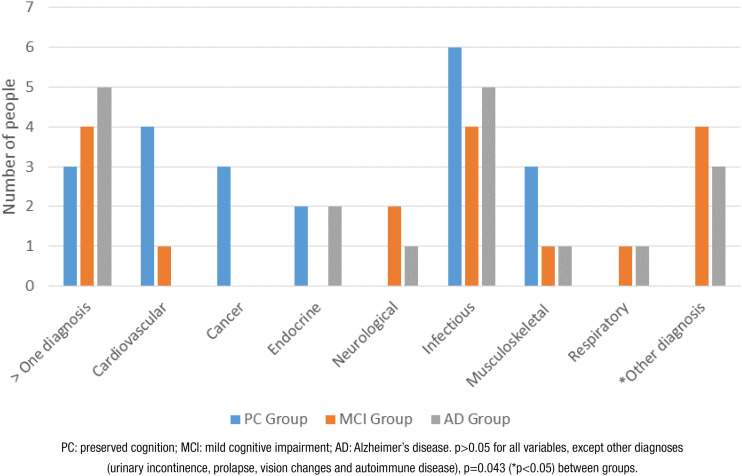
New diagnoses during follow-up.

Regarding new diagnoses, the participants reported new cardiovascular, cancer, endocrine, neurological, infectious, musculoskeletal and respiratory diseases as well as other diagnoses, such as urinary incontinence, prolapse, vision changes and autoimmune disease, which were grouped due to the low prevalence in the sample. The PC group did not have “other diagnoses”. All groups had new diagnoses of infectious diseases over the 32 months, with no significant difference in incidence between groups (Figure 2).

**Figure 3:**
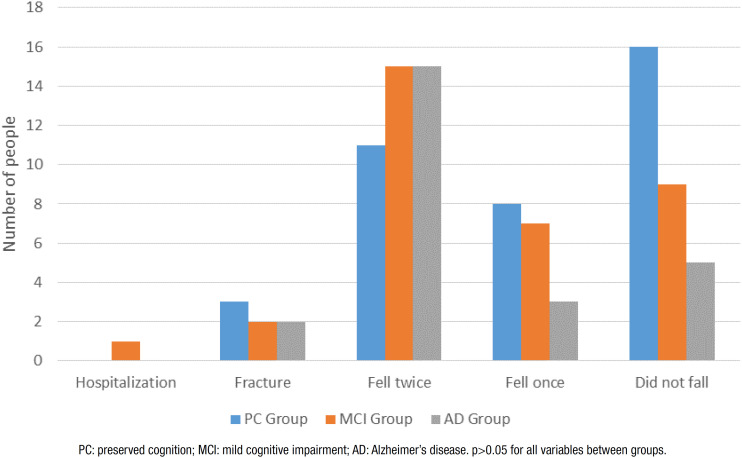
New falls and their consequences during follow-up.

**Figure 4:**
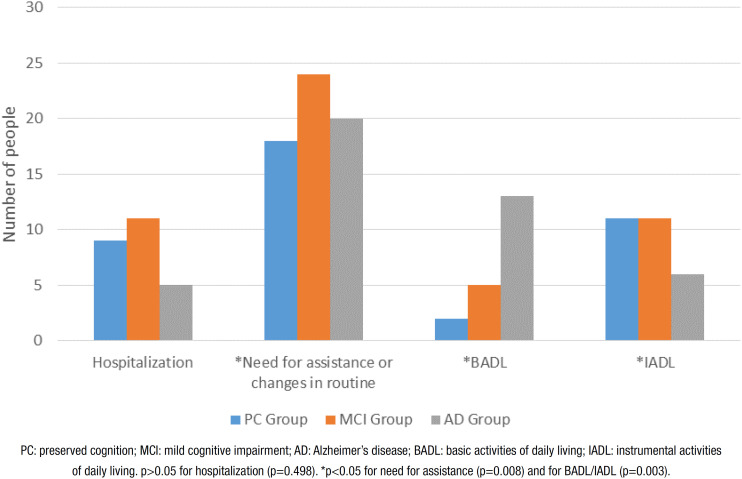
Hospitalization and need for assistance/changes in routine during follow-up.

No significant differences between groups were found regarding hospitalizations or the number and consequences of falls, although falls were more prevalent in the groups with cognitive impairment, especially the AD group. The need for assistance/changes in routine was higher in the AD group than the other groups, especially regarding basic ADLs (Figures 3 and 4).

No significant difference between groups was found regarding the use of medication, although higher frequencies were found in the groups with cognitive impairment. In the AD group, 56% of the participants increased their use of psychotropic drugs, compared to a prevalence of 35 and 25% in the PC and MCI groups, respectively (p>0.05). The groups also did not differ significantly regarding participation in social activities, physiotherapeutic interventions or the practice of physical exercise (Table 2).

**Table 2:** Interventions performed during follow-up.

Interventions, n (%)		PC group (n=35)	MCI group (n=31)	AD group (n=23)	p-value
Increased use of medication		7 (20)	13 (41.9)	11 (47.8)	0.055
	Number of increased medications, Md (IQR)^a^	1 (1–2)	1,0 (1–4)	2,0 (2–3)	0.219
	Use of psychotropic drugs, n (%)^a^	13 (35.14)	8 (25.81)	13 (56.52)	0.071
Participation in social activities		16 (45.7)	10 (32.3)	10 (43.5)	0.508
Type of social activity					
	Religious^b^	9 (60)	5 (50)	7 (70)	0.590
	Voluntary^b^	1 (6.7)	2 (20)	0 (0)	
	Cultural or education^b^	5 (33.3)	3 (30)	3 (30)	
Physiotherapy intervention		14 (40)	14 (45.2)	9 (39.1)	0.880
Physical exercise		20 (57.1)	15 (48.4)	7 (30.4)	0.135
Weekly frequency	
	Once^c^	1 (5)	2 (13.3)	0 (0)	0.135
	Twice^c^	5 (25)	6 (40)	1 (14.3)	
	Three times^c^	2 (10)	5 (33.3)	3 (43.9)	
	Four times or more^c^	12 (60)	2 (13.3)	3 (43.9)	
Type of activity					
	Aerobic^c^	10 (50)	7 (46.7)	5 (71.4)	0.971
	Water aerobics^c^	4 (20)	3 (20)	1 (14.3)	
	Gymnastics^c^	1 (5)	1 (6.7)	0 (0)	
	Stretching^c^	3 (15)	2 (13.3)	0 (0)	
	Strengthening^c^	2 (10)	2 (13.3)	1 (14.3)	

n (%): number of individuals (percentage); Md (IQR): median (interquartile range 25–75); PC: preserved cognition; MCI: mild cognitive impairment; AD: Alzheimer's disease;

a percentage of individuals who increased use of medication;

b percentage of individuals who participated in social activities;

c percentage of individuals who engaged in physical exercise.

## DISCUSSION

The AD group was older than other groups at baseline. This finding is consistent with data described in previous studies, which report that the incidence of dementia doubles every five years.^21^ The MCI group had a much higher proportion of women. Previous studies also report a greater number of older women with MCI.^22^ ^,^ ^23^ Moreover, the AD group had a lower calorie expenditure compared to the PC group, which is in line with data described by Pedroso et al.,^24^ who also found that older people with AD have low activity levels. Regarding the number of medications and diseases, higher numbers were found in individuals with cognitive impairment. Carvalho et al.^25^ report a higher frequency of two or more chronic diseases in older Brazilians, stating that this situation increases with age and is accompanied by a greater use of medications. Therefore, individuals with cognitive impairment use more pharmacological than non-pharmacological strategies.

In the present study, six individuals with cognitive impairment died during the follow-up period. In a previous longitudinal study with a six-year follow-up, the mortality rate of individuals with MCI was higher compared to those with PC.^26^ Furthermore, there is a progressive increase in mortality with advance in age.^2^ Interestingly, the average age of the individuals who died was 73.3 years (range: 65 to 85 years), which was similar to the average age of the survivors in the MCI and PC groups, as shown in Table 1.

After 32 months, the need for assistance/changes in routine was higher in the AD group, especially regarding basic ADLs. No significant differences among the groups were found regarding new diagnoses (except for “other diagnoses”), falls or hospitalizations. The most frequent new diagnoses were infectious and cardiovascular diseases in the PC group and infectious and “other diagnoses” in the MCI and AD groups. These results were expected, as the incidence of cardiovascular disease increases during the aging process^27^ and low-grade tissue changes and inflammation make older people more susceptible to infections, such as pneumonia.^28^ “Other diagnoses” included urinary incontinence, which is more common in individuals with cognitive impairment. This may be explained by the fact that functional, cognitive and motor changes in these patients lead to functional limitations when entering the bathroom.^2^ ^,^ ^29^

Although not statistically significant, falls were more prevalent in the groups with cognitive impairment, especially the AD group. In a longitudinal study with a six-month follow-up using a fall calendar and monthly phone calls, 52.6% of the individuals with MCI and 51.4% of those with mild AD experienced falls.^22^ The level of physical activity, participation in social activities and the supervision of caregivers can influence the exposure of older people to a variety of environmental stimuli that can exert an impact on the risk of falls. Moreover, the 32-month period increases the risk of recall bias and the prevalence of falls may actually be underestimated.

Regarding the need for assistance, the first activities to be compromised in AD are advanced and instrumental ADLs, followed by basic ADLs in advanced stages of the disease.^2^ Furthermore, previous cognitive decline predicts functional impairment in older people with mild AD.^30^ Thus, better knowledge regarding the evolution of AD and MCI can help family members and health professionals design care strategies to slow the progression of functional impairment.

In contrast to the present results, Rudolph et al.^31^ found that two-thirds of older people with mild AD were hospitalized at least once in a three-year period. Individuals with more accelerated functional decline prior to the diagnosis of dementia have a higher frequency of hospitalization. In another study, MCI was associated with a 17% increase in the hospitalization hazard index compared to normal cognition.^32^ Thus, MCI and AD should be target conditions for healthcare providers to reduce hospitalization rates.

In the present study, no significant difference was found between the groups regarding interventions in the follow-up period, but more than 40% of the individuals with MCI and AD increased their use of medications and 56% of those with AD increased their use of psychotropic drugs. Polypharmacy is associated with a worsening functional status even in early stages of dementia.^33^ Moreover, the use of psychotropic drugs increases the risk of falls in older people.^34^ Non-pharmacological interventions can be employed to control health problems in individuals with cognitive impairment and lower the need for pharmacological interventions.

Twenty-five percent of Brazilian older people with low participation in social activities have a low perception of social support, visual impairment, depressive symptoms, low cognitive status and loneliness, and are over 80 years of age.^35^ A lack of participation in social activities is an early indicator of negative health outcomes, as interacting with other individuals and the environment places more of a demand on cognitive faculties. Therefore, incentives are needed to maintain or increase social networks and consequently improve mental health and health behaviors, regardless of cognitive status.^36^

Physiotherapeutic treatment constitutes another alternative to pharmacological intervention. Physiotherapy can benefit individuals with AD and their families by improving cognition, psychiatric disorders, balance, functioning and quality of life as well as reducing the risk of hospitalization.^37^ In clinical practice, however, referrals to physiotherapy usually only occur in advanced stages of AD. Knowledge on the benefits of physiotherapy for all stages of AD should be disseminated among health professionals and the community.

Although not statistically significant, the PC group engaged in more physical exercise and had a higher weekly frequency of exercise than the other groups. Moreover, aerobic exercise was the most common type of activity practiced in all groups. Physical exercise is an important non-pharmacological intervention for older people^38^ and its practice at a regular frequency and moderate intensity is associated with a decreased incidence of AD as well as fewer risk factors and falls.^39^ Other positive effects include neuronal plasticity, neurogenesis, improvements in cognition and vascular function and a reduction in inflammation.^40^ Aerobic exercise improves cognitive performance in older people. When combined with strengthening exercises, aerobic exercise benefits working memory, attention and processing speed, especially in people with MCI.^41^ The effects of physical exercise are influenced by prior participation in social activities among older people and, when combined, these interventions can reduce the risk of disability by 57%.^42^ Thus, regardless of cognitive and functional impairment, the regular practice of physical exercise and social activities should be stimulated in older people.

Despite the relatively under-explored approach of analyzing clinical outcomes and interventions in older people in the initial stages of cognitive impairment, this study had some limitations that should be considered: the convenience sample, the small sample size, the application of the follow-up questionnaire through home visits rather than by telephone in some cases and the fact that some clinical outcomes may have not been reported by informants or volunteers due to forgetfulness. Despite this, the questions were directed at the nearest informant of individuals with cognitive impairment to minimize the possibility of bias. We were careful to define falls during the interviews. However, this information was collected after 32 months. Other instruments, such as a fall calendar and monthly telephone calls, could have quantified this event better. Future longitudinal studies involving clinical outcomes and interventions in older people with and without cognitive impairment with a shorter follow-up period are needed. Moreover, future research addressing the transition of the cognitive diagnosis (MCI to AD) over time is important for a better understanding of the evolution of cognitive impairment. Finally, information should be centralized, and better communication among health professionals is needed regarding the needs of older people, caregivers and family members to enable the development of comprehensive, multidimensional care strategies.

In conclusion, significant differences between older people with PC, MCI and AD were found regarding the need for assistance or change in routine and new diagnoses of specific diseases after a 32-month period, whereas no significant differences were found in the occurrence of deaths, falls, hospitalizations or different interventions. Health professionals need to be aware of different clinical outcomes and interventions and must take into account the multidimensionality of geriatric patients when planning assessments and interventions.
